# Children and HIV – a hop (hopefully), a skip (lamentably) and a jump (ideally)?

**DOI:** 10.1080/09540121.2016.1176688

**Published:** 2016-07-08

**Authors:** Marie-Louise Newell, Ashraf Grimwood, Lucie Cluver, Geoffrey Fatti, Lorraine Sherr

**Affiliations:** ^a^ Southampton University, Southampton, England; ^b^ Kheth’Impilo, Cape Town, South Africa; ^c^ Centre for Evidence-Based Interventions, Department of Social Policy & Intervention, University of Oxford, Oxford, UK; ^d^ Department of Psychiatry and Mental Health, University of Cape Town, Cape Town, South Africa; ^e^ Department of Infection and Population Health, University College London, London, UK

The noted children’s human rights lawyer, Michael Freeman, posed the provocative question of whether the world viewed children as “beings or becomings?” (Freeman, 2015). This encapsulates some of the challenges and dilemmas in the way children infected or affected by HIV are served. Narratives around “investing for the future” and “tomorrow’s generation” are in line with an idea that children are becoming adults, becoming human – but obscure their current needs and understate the place of children as current active agents.

An equity focus, as called for in this special issue of *AIDS Care*, would support the needs of children as beings. The end of 2015 saw the end of the Millennium Development Goals (MDGs), and achievements inclusive of substantial reduction of mother-to-child transmission (MTCT) of HIV, prevention of HIV and decrease in HIV incidence as well as expansion of HIV treatment and care programmes globally, but in particular in the most-affected countries of Sub-Saharan Africa (SSA). Yet there are situations where outcomes for children are wanting. These include the unacceptably high 30% MTCT in the Democratic Republic of the Congo (Edmonds et al., 2015; United Nations General Assembly Special Session [UNGASS], 2014) where decentralisation has not ensured higher proportions of pregnant women receiving the full package of interventions at antenatal care, Zimbabwe struggling to reduce national vertical transmission rates to under 5%, being 9.6% in 2013 (UNAIDS, 2014a) and Burundi’s modelled at 24.5% (UNGASS, 2015). Children continue to lag in the equity response, with, compared to adults, fewer children on treatment, 76.9% of adults versus 46% of children in Zimbabwe and 12% in Burundi (UNAIDS, 2014a). It is also notable that fewer children are tested for HIV, and that children receive consistently fewer mentions or considerations in plans, policies and future agendas (Sherr, Cluver, Tomlinson, & Coovadia, 2015). Even those organising the 2016 high-level UN meeting's civil society consultations forgot that children were part of civil society. Newer antiretroviral treatment (ART) drugs are not formulated for children, making administration and ultimately adherence even more of a challenge. Young girls remain disproportionately affected by HIV compared to boy children (Singh, Rai, & Kumar, 2013).

In 2014, 220,000 (190,000 in SSA) children below the age of 15 years were living with HIV globally, nearly all of whom became infected through MTCT. The prevalence of HIV among pregnant women varies from less than 1% in resource-rich settings of Western Europe, America and Asia to over 40% in some areas of southern Africa (UNAIDS, 2014b). Although the UN has prioritised the virtual elimination of vertically-acquired HIV, the complex machinery and interplay of policy, implementation and review needs to be further expanded for this final goal to be met – achievable as evidenced by the dramatic downturn of children infected at birth as recorded in many settings.

By March 2015, 15 million people had been initiated onto ART globally, nearly 11 million in SSA; it is unclear how many remain on treatment and how many of these are virally suppressed. About 40% of HIV-infected adults were estimated to have accessed ART, but only 32%, or even less, of children; in 2014 nearly three-quarters of pregnant women living with HIV had access to ART for the prevention of mother-to-child transmission (PMTCT) – again high numbers although universal access is not yet enjoyed everywhere. Elimination would require a complete cascade of programmatic provision to ensure universal and appropriately regular HIV testing of all pregnant women to ensure identification of HIV early in pregnancy, followed by comprehensive roll out of appropriate HIV care, ART and support. Better still, not only should HIV-negative pregnant women in high-prevalence areas be tested regularly, but also their households including their partners – this has been reported to halve HIV acquisition (Fatti, Ngonzo, & Grimwood, 2013) in these most vulnerable women. The 2010 WHO PMTCT B+ regime, which included sustained ART for life for pregnant or lactating women once diagnosed, contributes to preventing MTCT during pregnancy and postnatally and reduced transmission to sexual partners – thus indirectly protecting fatherhood as well. WHO recommendations relating to treatment eligibility have been expanded over time, with the most recent, September 2015, guidelines suggesting starting ART immediately upon HIV diagnosis for all, irrespective of clinical or immunological disease progression. This new recommendation thus merges PMTCT with optimal treatment of HIV-infected women. And now there is pre-exposure prophylaxis which could be a valuable adjunct to safer/barrier sex in further protecting women and reducing HIV transmission in discordant couples. All these elements can contribute to provision for safe and healthy families – the bedrock of child development and positive child environments.

MTCT can occur before, during and after delivery. With the use of ART in the prevention of MTCT, rates of MTCT have come down substantially and – where PMTCT is available and effective – are now less than 1% at six weeks of age (reflecting peripartum acquisition of infection) and <4–5% at 18 months of age (depending on the duration of breastfeeding under the cover of ART); it is anticipated that with the further expansion of ART for treatment and prevention, rates at the end of breastfeeding will be below 2% – already <1% has been achieved in some of the busier clinics in the KwaZulu-Natal midlands of South Africa. Community support for pregnant women has also shown promise in improving PMTCT outcomes (Fatti, Shaikh, Eley, & Grimwood, 2016). With PMTCT and ART for life for the mother, child mortality rates have declined substantially; mortality rates in uninfected children born to mothers with HIV who are on ART are similar to those in children born to HIV-uninfected mothers, reflecting the increase in maternal survival benefit which extend to all her children.

These varying health outcomes highlight the complex multidimensional facets of inequity – where the interplay of poor social justice, the lack of children’s right and ethics result in the inequalities in health in most countries which are unnecessary, preventable and unjust (Singh et al., 2013).

Preventing HIV infection in infants is a primary challenge; yet there is more to the needs of children than PMTCT or ART for those infected. To thrive, they need to be born into a family with good parenting, parents who both survive and lead productive lives to ensure economic and social stability. The importance of the early child years for optimal development and later health is undoubted, with researchers highlighting the need to invest in the first 1000 days of life from conception (Engle, Castle, & Menon, 1996; Richter & Naiker, 2013; Sherr, 2005). HIV in the family has the propensity of disrupting and dismantling many of the pillars of early child provision, exacerbating inequity, leading to further inequality and spiralling the household into secondary poverty where there is no social service safety net or adequate healthcare provision. Society needs to ensure the first 1000 days are well supported for optimum health and social outcomes for the child and parents, as reflected in the Sustainable Development Goals (SDGs). Certainly society could do well to extend this to the first 1000 weeks of life – bringing newborns safely to adulthood in those 19 years.

ART for HIV-infected adults has considerably improved life expectancy which will directly affect parenting and the quality of early years care for infants. Life expectancy for adults in rural South Africa has increased from a nadir of 49.2 years in 2003, before the introduction of ART for HIV-infected adults to 60.5 years in 2011, five years after the start of the HIV treatment and care programme in the area (Bor, Herbst, Newell, & Bärnighausen, 2013). The more extensive the treatment coverage of HIV, the lower the incidence: if ART coverage increases to over 20%, the rate of new HIV infections in the same population decreases by 23% and by 38% for over 30% (Tanser, Bärnighausen, Grapsa, Zaidi, & Newell, 2013); the idea forms the basis for the UN Fast Track goals of 90% of people HIV-infected knowing their status and 90% of these accessing ART with 90% of these being virally suppressed, as a stepping stone to reaching 95% for each of these by 2030 in anticipation of an AIDS-free generation. Yet beyond birth there is the need to support caregivers to be carers. It is well established that the quality of early caregiving dramatically effects child development outcomes with long-reaching effects (Walker, Chang, Vera-Hernández, & Grantham-McGregor, 2011; Walker, Chang, Wright, Osmond, & Grantham-McGregor, 2015; Walker, Chang, Younger, & Grantham-McGregor, 2010). Caregiver interventions need to be integrated into provision (Chang et al., 2015).

Before the widespread use of ART, HIV infection was shown to reduce fertility, with live birth rates in HIV-infected women generally about half of those of HIV-uninfected women. However, with the expansion of HIV treatment and care programmes and availability of ART, childbearing decisions now take place in a different context than was previously the case. With ART, an increase in fertility has been reported in some, but not all, studies, with levels nearing those of HIV-uninfected women. It is currently unclear what would drive an increase in fertility after ART initiation, but in qualitative research, women suggested that their childbearing decisions were not influenced directly by their HIV treatment, but by personal circumstances, in particular, their relationship with their partner, but also concerns about their health, their future and the fact that the chances of having a healthy negative child touching 100%, similar to a woman HIV-negative.

Although the success in prevention of MTCT has been impressive, with Cuba declared to have eliminated vertically-acquired HIV infection by WHO (2015) and the world now starting to talk about eMTCT for elimination of MTCT, HIV treatment and care programmes face considerable challenges. These challenges range from the need for primary HIV prevention in women of childbearing age, particularly relevant, considering the continued high HIV incidence amongst young women in SSA (Harrison, Colvin, Kuo, Swartz, & Lurie, 2015); high rates of unintended pregnancy in HIV-affected communities (Phillips & Mbizvo, 2016) with, for example, 14% of high school girls in rural KwaZulu-Natal falling pregnant annually (unpublished data, Shaikh, 2016), the intersecting epidemics of alcohol use, interpersonal violence and HIV infection in pregnant women (Russell, Eaton, & Petersen-Williams, 2012), late presentation of pregnant women for antenatal care (du Plessis et al., 2014), inadequate uptake of the full cascade of PMTCT services for HIV-infected pregnant women (Woldesenbet, Jackson, Lombard, et al., 2015), a high incidence of late gestational and postpartum HIV acquisition that significantly contributes to paediatric HIV (Johnson et al., 2012) and important individual and contextual barriers to ART initiation, adherence and retention in pregnant women (Hodgson et al., 2014). Thus, coverage with ART to delay disease progression in pregnant woman and to prevent MTCT remains suboptimal in many if not most settings. Further, there is a big drop in follow-up within programmes post-delivery, even though continued ART is essential to prevent MTCT through breastfeeding and for maternal health. In addition, despite improvements in recent years, routine early infant diagnosis for HIV-exposed infants remains suboptimal (Woldesenbet, Jackson, Goga, et al., 2015), which has resulted in delays in ART initiation for HIV-infected infants who frequently start ART with severe HIV disease (Porter et al., 2015). These factors reflect the inequity facing these communities – over 60% unemployment in rural South Africa, coupled with food, shelter and personal insecurity, lack of basic services such as water and sanitation. Not addressing these will preclude optimal health outcomes for children.

Much of the treatment debate surrounds child survival questions, but by this point, the focus on child thriving has moved centre stage. The transition from the MDGs to the SDGs clearly illustrates this more holistic and comprehensive imperative. This occurs at the very time that resources are less available, priorities are torn in many directions and the demands are high. The needs of children have moved from an equality to an equity call. There are specific groups who are more vulnerable – there are specific needs that may require disproportionate investment and there are specific interventions that will have to be prioritised and resourced. Equity moves us beyond the essential survival aspects of PMTCT, to consider the lives of children and adolescents in HIV-affected families. For those children and adolescents who are HIV-positive, research identifies low adherence to ART (Nachega et al., 2009), cognitive limitations (Sherr, Croome, Bradshaw, & Parra Castaneda, 2014) and stigma (Mburu et al., 2014). For others who are uninfected, challenges include bereavement, the need to provide care for unwell family members in situations of extreme poverty (Skovdal, 2010), stigma-by-association (Boyes, Bowes, Cluver, Ward, & Badcock, 2014) and strong situational drivers of risk behaviours. One of the greatest equity concerns is that children in HIV-affected families are themselves at increased likelihood of HIV infection – their vulnerability leading to intergenerational risk.

HIV presents multiple and severe challenges to equity for children. This special issue was commissioned to revisit this equity theme. At the International AIDS Society conference in Durban in 2000, President T. Mbeki missed an important opportunity to link inequity and HIV. He said at the opening plenary that poverty was the cause of AIDS and not a virus, what he should have said was poverty increases the susceptibility to infection and the impact of the infection on the health of the individual as well as their family and that HIV also exacerbates poverty. The Malthusian approach that characterised the government at the time saw many preventable infections and deaths.

The UN Fast Track goals form the basis of how the submitted articles for this special edition are grouped. The first group of papers look at testing and diagnosis of HIV (the first 90%), the second cluster (second 90%) on linkage to care and treatment and the third cluster on adherence and viral suppression. The editors have also created a fourth category that the majority of papers talk to that encompasses the continuum of care incorporating the psychosocial aspects of HIV.

The chosen papers indicate various challenges for which interventions need to be developed or improved to ensure all HIV-affected children “survive, develop and reach their full potential without discrimination” (UNICEF, 2011). It gathers together papers that explore resilience, geographical areas of high burden and high need and explores a number of elements of the care cascade for children. Five papers examine the importance of caregiver issues, four comment on adherence to treatment – vital for efficacious long-term viral suppression and quality survival, two focus on MTCT, two papers examine issues of HIV testing and counselling and five look in-depth at those with HIV (parents *n* = 2 and children *n* = 3). The selection is not exhaustive and coverage not comprehensive, but this issue gives insight into current areas of research, concern, understanding and achievement from a wide geographical area. The majority of studies are carried out in SSA, reflecting the epicentre of child HIV burden, but papers from China, Haiti and Panama were also included to demonstrate and reflect the global challenges. Wei, Li, Harrison, Zhao, and Zhao (2016) examine the psychological factors that reduce the effects of HIV stigma and promote resilience in HIV-affected children in China. Barenbaum and Smith (2016) use the Social Action Theory framework to look into the elements that contribute to the positive development and resilience of HIV-positive adolescents – giving insight into why HIV-positive children have a lower than expected rate of mental health problems in South Africa. Sharer, Cluver, Shields, and Ahearn (2016) explore the relationship between family-social support and mental health disorders and elaborates on whether family-social support acts as a source of resilience for HIV-affected adolescents in South Africa. Chaudhury et al. (2016) note that HIV-affected families often have higher rates of harmful alcohol use, intimate partner violence and family conflict in Rwanda. Her paper proposes a family intervention to reduce these occurrences which are detrimental to the child’s well-being. Barenbaum and Smith (2016) highlight the importance of trusting caregivers for the well-being of children suffering parental loss and discuss social support interventions. Goldberg and Short (2016) explore whether Kenyan mothers in HIV-affected households were willing to foster homeless children. HIV can disrupt functioning in many ways and the paper by Skovdal (2016) explores why HIV-affected children have classroom concentration problems and consequently poorer educational outcomes. Hensels et al. (2016) focus on gender differences in children attending community-based organisations in Malawi and South Africa, highlighting that for younger children there are many similarities across the gender divide, but when there are differences, it is boys who are disadvantaged and overlooked – perhaps prompting the equity needs of boys to be maintained, although girls have often been the target of interventions. In a systematic review, Goldberg and Short (2016) summarise the current literature on children living with HIV-infected adults and offer suggestions for future research in this area. The effectiveness of ART requires life-long adherence, and five papers investigate why adherence levels are low in HIV-positive individuals. Estripeaut et al. (2016) examine in Panama the cultural and psychosocial factors that influence adolescents’s failure to take their medication from a gender perspective. Bermudez et al. (2016) argue that the economic and social circumstances of youth living with HIV in Uganda affect their adherence to antiretroviral therapy. Cluver et al. (2016) assess social protection provisions, and demonstrate how the provision of cash plus care is associated with significantly enhanced adherence. Coetzee, Kagee, and Bland (2016b) comment on the insufficient adherence counselling given to caregivers of children receiving ART in rural South Africa and highlight the need for improved training and support provided to counsellors. These studies show the importance of quality provision, yet Coetzee, Kagee, and Bland (2016a), utilizing video-recording of adherence counselling, pick up the inadequacies of these encounters which last for less than 8 min, contain very little – if any – counselling and rely on lay workers who may not provide adequately for effective adherence in children under five who are reliant upon “mediated adherence” as their caregivers administer treatment. HIV counselling and testing is believed to be inadequate for HIV-exposed children; Olaleye et al. (2016) explore the perspective of the health workers who provide paediatric HIV counselling and testing. Thurman, Luckett, Taylor, and Carnay (2016) report on the impact that home visits by community-based care workers have on the likelihood of a high-risk orphaned child being tested for HIV. Busza et al. (2016) focus on female sex workers and described workshops to encourage clinical service uptake in Zimbabwe. Following this, Fiori et al. (2016) write a short report on the strategies that need to be implemented to optimise paediatric HIV care delivery in Togo, West Africa. Chamla et al. (2016) comment on caregiver satisfaction with paediatric HIV treatment in Nigeria, where satisfaction with the availability of services and attitude of health workers was measured. Lastly, the paper by Kidman and Heymann (2016) focuses on caregivers, presenting a conceptual framework to help address the needs of HIV-affected caregivers. Her paper also examines the availability of social policies in 25 highly affected countries in SSA.

These papers feed into the Coalition on Children affected by HIV/AIDS meeting at Durban 2016, in a concerted effort to keep attention and energy focussed on the broad and ongoing needs of children. The challenges come from many directions. As gains in treatment and care are made, the spotlight needs to be on the updating of treatment guidelines, health policies, improving implementation and the review of these as well as health care system and community support systems strengthening. Improved linkages between health services and the community are required. Community-support programmes, in which psychosocial challenges in households of HIV-affected children are identified and addressed, have been shown to improve outcomes for children receiving ART and improve early childhood development outcomes (Fatti, Ngonzo et al., 2013; Fatti, Shaikh, Eley, & Grimwood, 2013; Grimwood et al., 2012). Human behaviour must be understood and accommodated so that disclosure, adherence and cascade shortcomings can be prevented. The contexts and worlds of children matter, and young children are particularly reliant on advocates to protect their interest, fight for their rights and provide for their needs. Children are not *becomings* – they are *beings*. Yet with their being underserved, mistreated and overlooked, they are becoming impatient. True progress can only be measured by the extent to which equity for children has been achieved universally. The UN fast track targets can be used as proxy measures to monitor equity being achieved as we head towards the 2020, 90-90-90 goals on the road to the 2030 goals of an AIDS-Free Generation.
